# Multi-UAV Escape Target Search: A Multi-Agent Reinforcement Learning Method

**DOI:** 10.3390/s24216859

**Published:** 2024-10-25

**Authors:** Guang Liao, Jian Wang, Dujia Yang, Junan Yang

**Affiliations:** 1 College of Electronic Engineering, National University of Defense Technology, Hefei 230037, China; liaoguang23@nudt.edu.cn (G.L.); yangdj@nudt.edu.cn (D.Y.); yangjunan@ustc.edu (J.Y.); 2 Anhui Province Key Laboratory of Electronic Restriction, Hefei 230037, China

**Keywords:** multi-UAV, area coverage path planning, escape target search, multi-agent reinforcement learning

## Abstract

The multi-UAV target search problem is crucial in the field of autonomous Unmanned Aerial Vehicle (UAV) decision-making. The algorithm design of Multi-Agent Reinforcement Learning (MARL) methods has become integral to research on multi-UAV target search owing to its adaptability to the rapid online decision-making required by UAVs in complex, uncertain environments. In non-cooperative target search scenarios, targets may have the ability to escape. Target probability maps are used in many studies to characterize the likelihood of a target’s existence, guiding the UAV to efficiently explore the task area and locate the target more quickly. However, the escape behavior of the target causes the target probability map to deviate from the actual target’s position, thereby reducing its effectiveness in measuring the target’s probability of existence and diminishing the efficiency of the UAV search. This paper investigates the multi-UAV target search problem in scenarios involving static obstacles and dynamic escape targets, modeling the problem within the framework of decentralized partially observable Markov decision process. Based on this model, a spatio-temporal efficient exploration network and a global convolutional local ascent mechanism are proposed. Subsequently, we introduce a multi-UAV Escape Target Search algorithm based on MAPPO (ETS–MAPPO) for addressing the escape target search difficulty problem. Simulation results demonstrate that the ETS–MAPPO algorithm outperforms five classic MARL algorithms in terms of the number of target searches, area coverage rate, and other metrics.

## 1. Introduction

In recent years, unmanned aerial vehicles (UAVs) have found applications in various military and civilian domains due to their advantages, such as high mobility, accessibility, convenient deployment, and low cost. They have gradually become indispensable in modern society, with roles in civil sectors, such as agriculture [1,2], mineral exploration [3], and forest rescue [4], as well as in military reconnaissance [5] and strikes [6]. Multi-UAV target search problems is a significant issue in autonomous UAV decision-making and has garnered extensive academic attention recently. Multi-UAV target search involves UAVs using on-board detection equipment to reconnoiter designated areas. They share information via a communication network, thereby jointly capturing targets. Currently, three primary methods are used for multi-UAV target search. The first category is pre-planning methods, such as partition search [7] and formation search [8]. These methods, essentially, transform the target search problem into a planning problem with area coverage, offering high reliability and easy evaluation of the solution results. However, they require a known mission area model in advance, involve longer planning times, and are not highly adaptive to dynamic environmental changes. The second category is online optimization methods, which approximate the search problem as a real-time objective function optimization problem. These methods typically employ traditional or heuristic algorithms, such as ant colony algorithms [9] and genetic algorithms [10]. They are better adapted to environmental dynamics than to pre-planning approaches. However, they depend on a central node for decision-making and exhibit low adaptability in distributed environments. The third category entails Multi-Agent Reinforcement Learning (MARL) methods, which model the problem as a Partially Observable Markov Decision Process (POMDP) and utilize algorithms based on the MARL framework. These methods enable agents to learn and optimize their behavior through interactions with the environment and other agents, allowing them to adapt to dynamic changes and make rapid decisions [11,12]. The primary challenge of these methods lies in designing the algorithm training architecture, agent exploration mechanism, and reward function tailored to specific task requirements.

Recently, the design of MARL methods has become a prominent area of research in the field of artificial intelligence. It has found applications in areas such as multi-UAV target search [13], autonomous vehicle path planning [14], and other related applications. Within the MARL framework, Shen et al. [15] proposed the DNQMIX algorithm, which enhances search rate and coverage. Lu et al. [16] proposed the MUICTSTP algorithm, demonstrating superior performance in terms of anti-interference and collision avoidance. Yu et al. [17] proposed the Multi-Agent Proximal Policy Optimization (MAPPO) algorithm, which has exhibited excellent performance in multi-agent testing environments and is regarded as one of the most advanced algorithms available. Wei et al. [18] combined the MAPPO algorithm with optimal control (OC) and GPU parallelization to propose the OC–MAPPO algorithm, which accelerates UAV learning.

To better describe environmental uncertainty, Bertuccelli et al. [19] proposed a probabilistic approach. This method divides the task area into units, each associated with the probability of target presence, establishing a target probability graph. The method has achieved good results in target search and is widely recognized. Building on the MARL framework and the target probability graph, Zhang et al. [20] designed a confidence probability graph using evidence theory and proposed a double critic DDPG algorithm. This approach effectively balances the bias in action–value function estimation and the variance in strategy updates. Hou et al. [21] converted the probability function into a grid-based goal probability graph and proposed a MADDPG-based search algorithm, improving search speed and avoiding collisions and duplications.

Multi-UAV target search has made some progress over time, but two challenges remain. Firstly, the utilization of sample data remains inefficient, and balancing utilization and exploration presents a challenge. Existing MARL algorithms primarily employ neural networks, such as fully connected networks and convolutional networks. These networks often fail to simultaneously achieve efficient utilization of temporal and spatial information in the sample data, and also lack effective environmental exploration. Secondly, the behavioral modeling of dynamic target is relatively simple. Existing work primarily considers changes in the target’s position over time, often transforming the target search problem into a target tracking problem. In actual non-cooperative target search scenarios, targets may exhibit escape behavior. They actively change their position to evade detection and, potentially, use environmental blind spots to hide, preventing real-time tracking by UAVs. Addressing the challenges identified above, this paper investigates the Multi-UAV Escape Target Search (METS) problem in complex environments. The contributions of this paper are summarized as follows:

1. The simulation environment for the METS problem is constructed, introducing a Target Existence Probability Map (TEPM), with an appropriate probability update method employed for the escaping target. Based on the TEPM, a local state field of view is designed, with local state values obtained through entropy calculation. Additionally, a new state space, action space, and reward function are devised within the framework of Decentralized Partially Observable Markov Decision process (DEC-POMDP). Ultimately, a model that addresses the METS problem is established.
2. To enhance the MARL algorithm’s ability to process spatio-temporal sequence information and improve environmental exploration, this paper proposes the Spatio-Temporal Efficient Exploration (STEE) network, constructed using a convolutional long short-term memory network and a noise network. This network is integrated into the MAPPO algorithm, and its impact on the overall performance of the MAPPO algorithm is validated.
3. To search the escaping target in the METS problem, the Global Convolutional Local Ascent (GCLA) mechanism is proposed. A Multi-UAV Escape Target Search algorithm based on MAPPO (ETS–MAPPO) is introduced by combining MAPPO with the STEE network. This algorithm effectively addresses the challenges of searching for escape targets. Experimental comparisons with five classic MARL algorithms show significant improvements in the number of target searches, area coverage rate, and other metrics.

The remaining chapters of this paper are organized as follows: Section 2 defines the system model and provides a mathematical formulation of the METS problem. Section 3 introduces the ETS–MAPPO algorithm within the MARL framework and describes it in detail. In Section 4, experiment results are presented to validate the effectiveness of ETS–MAPPO. Section 5 concludes the paper and discusses future research.

## 2. System Model and Problem Formulation

### 2.1. System Model

**Figure 1:**
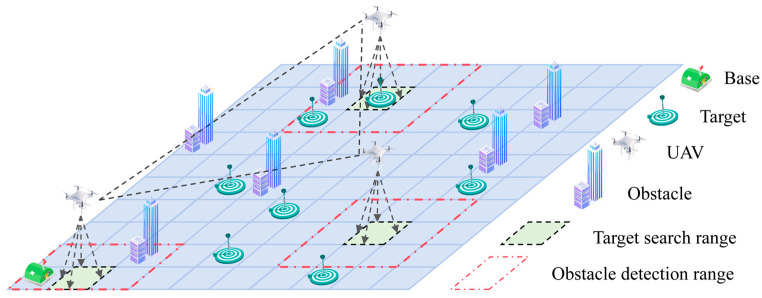
Typical scenario diagram for a multi-UAV escape target search mission.

A typical multi-UAV escape target search mission scenario is shown in Figure 1. Assuming that several escape targets and fixed obstacles are distributed within an unknown mission area Ω, $N_{u}$ UAVs depart from the base and cooperatively search the area. The mission requires UAVs to capture as many escape targets as possible, maximize coverage of mission area Ω, and avoid collisions with obstacles to the greatest extent possible.

#### 2.1.1. Mission Area Model

Considering the need for relative stability in the operation of the UAV detection payload, the UAV was set to maintain a constant flight altitude during target search operations in the mission area Ω [22]. In this scenario, the mission area Ω was conceptualized as a two-dimensional plane with two layers, where the UAV operated in the upper layer. Escape targets were positioned on the ground in the lower layer of the mission area. Obstacles were distributed across both the upper and the lower layers.

**Figure 2:**
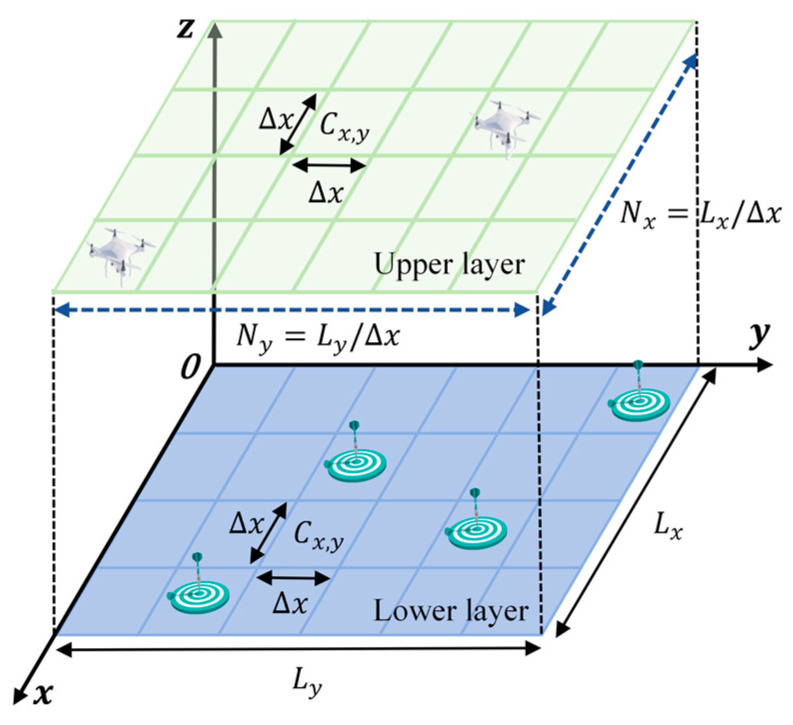
Rasterized model map of the task area.

In Figure 2, each layer is a rectangular region $L_{x}\times L_{y}$, which is rasterized into a cellular grid $N_{x}\times N_{y}$, with each cell having an area of S=Δx×Δx. The positional coordinates of any cell $C_{x,y}$ in the Cartesian coordinate system are denoted by Equation (1):

(1) $$C(x,y)=\{(x,y)|x\in [1,\;N_{x}],y\in [1,\;N_{y}]\}$$

#### 2.1.2. Unmanned Aerial Vehicle Model

**Figure 3:**
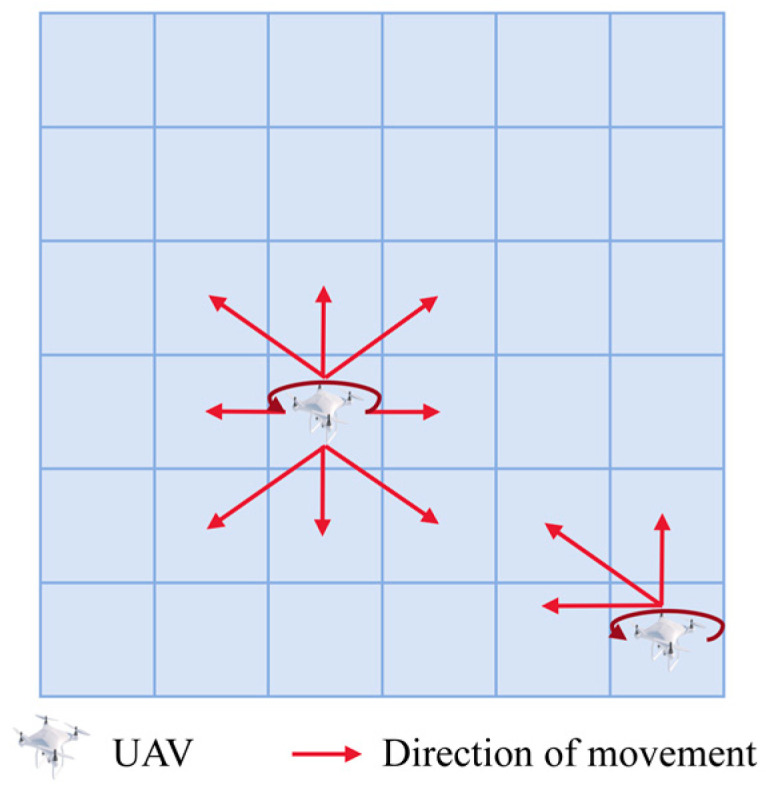
UAV action space model.

To facilitate the study, the motions of $N_{u}$ identical quadrotor UAVs were modeled as depicted in Figure 3. The UAVs were capable of discrete movements with a total of 9 degrees of freedom: left up, up, right up, left down, down, right down, left, right, and stationary.

The UAVs were equipped with a detection payload for target identification, with a detection range of $L_{target}$. In the rasterized mission area model, this detection range was mapped to $n_{u}=L_{target}/S$ cells. Assuming no omissions or false detections by the detection payload, it was considered that the UAVs could correctly identify a target within a cell at time t with a probability p. The UAVs’ detection range for stationary obstacles was $L_{obstacle}$, which was mapped to $n_{o}=L_{obstacle}/S$ cells.

#### 2.1.3. Escape Target Model

In the METS mission, the mission area contained $N_{t}$ escape targets, each of which could be in one of two states: stationary or escaping. These targets could detect UAVs within a detectable range of $L_{uav}$,which was mapped to cells $n_{t}=L_{uav}/S$. Initially, each escape target remained stationary, meaning that it maintained its current position while detecting UAVs. When a UAV entered the detection range of an escape target, the escape target transitioned from the stationary state to the escaping state. In the escaping state, the escape target engaged in active concealment, released false information to the UAV, and chose a movement distance $L_{chang}$ in a random direction with probability $P_{c}$, mapped to $n_{c}=L_{change}/S$ cells, or remained stationary with probability $1-P_{c}$, within the escape time step $T_{change}$. The escape target returned to the stationary state at the end of the escaping state.

#### 2.1.4. Target Existence Probability Map Model

In the METS problem, at the start of each search cycle, a probability distribution function was employed to model the target’s location information. In the rasterized mission area, the probability function was transformed into a cell-based Target Existence Probability Map (TEPM), with each cell having an associated target existence probability $b_{x,y}\in [0,1]$. The initial target existence probability for all cells in the TEPM was set to $b_{x,y}(0)=0.5$, indicating that the UAVs lacked any a priori information about target presence in the mission area.

As the UAVs continuously searched the mission area, their detection payload scanned for target information along the path, and the TEPM was probabilistically updated based on this detection information. We used a Bayesian update model to update the TEPM. When the UAV scanned cell $C_{x,y}$ at time t, the probability update for the target appearing in cell $C_{x,y}$ was given by Equation (2) [23]. If multiple UAVs scanned cell $b_{x,y}$ simultaneously, the probability value was updated correspondingly for each scan.

(2) $$b_{x,y}(t)=\frac{p_{s}b_{x,y}(t-1)}{p_{s}b_{x,y}(t-1)+(1-p_{s})(1-b_{x,y}(t-1))}$$

where $p_{s}$ is the correct probability p of the UAV’s detection payload in detecting a target in cell $C_{x,y}$. If no targets existed, $p_{s}$ was substituted with 1−p.

### 2.2. Formulation of the Problem

In the METS mission, the UAVs began at a base position and made operational decisions based on state information, aiming to explore the unknown mission area and search for the escape targets as extensively as possible. Consequently, the objective function was defined to maximize the exploration degree $f_{1}$ of the entire region while also maximizing the number of target searches $f_{2}$, as represented in Equation (3):

(3) $$\left\{ \begin{array}{l} f_{1}=\sum_{x=1}^{N_{x}}\sum_{y=1}^{N_{y}}\left|b_{x,y}(t)-p_{\max}\right|\\ f_{2}=\sum_{x=1}^{N_{x}}\sum_{y=1}^{N_{y}}1,b_{x,y}(t)>\varepsilon \end{array}\right.$$

where ε is the confidence level of the target’s existence in a cell and $p_{\max}$ is the maximum uncertainty value.

Considering that the UAV motion was constrained by the mission area’s boundaries, the boundary condition for the UAV at position $u_{i,t}(x,y)$ was expressed in Equation (4):

(4) $$\left\{ \begin{array}{l} 1\leq u_{i,t}(x)\leq N_{x},\forall u\in U\\ 1\leq u_{i,t}(y)\leq N_{y},\forall u\in U \end{array}\right.$$

Additionally, it is essential to consider that the UAV should avoid collisions with obstacles, with the collision constraint expressed in Equation (5):

(5) $$\left\|B_{k}(x,y)-u_{i,t}(x,y)\right\|>d_{safe},k\in \{1,2,\ldots ,N_{B}\}$$

where $B_{k}(x,y)$ is the position of obstacles k, $d_{safe}$ is the safety distance, and $N_{B}$ is the number of obstacles.

The collision constraint was subsequently transformed into the optimization objective of the objective function $f_{3}$, as expressed in Equation (6):

(6) $$f_{3}=\sum_{i=1}^{N_{u}}\sum_{k=1}^{N_{B}}1,\left\|B_{k}(x,y)-u_{i,t}(x,y)\right\|\leq d_{safe}$$

Finally, the METS problem was formulated as an optimization problem with an objective function, as represented in Equation (7):

(7) $$\begin{array}{l} \max\;\;f_{1}+f_{2}-f_{3}\\ s.t.\;\;\;1\leq u_{i,x}(t)\leq N_{x},\forall u\in U\\ \;\;\;\;\;\;\;\;\;1\leq u_{i,y}(t)\leq N_{y},\forall u\in U \end{array}$$

## 3. Multi-UAV Escape Target Search: MARL Approach

In this section, the optimization problem presented in Equation (7) is first reformulated within the framework of DEC-POMDP based on MARL methods. The state space of UAVs and the reward function are then defined according to the TEPM. Finally, the ETS–MAPPO algorithm is proposed, and its framework is detailed.

### 3.1. Decentralized Partially Observable Markov Decision Process

In the METS, an extended form of the POMDP was presented as the DEC-POMDP to model the optimization problem [24], considering multiple UAVs and decentralized decision-making. The DEC-POMDP is defined by the tuple $\left(N,O,A,S,F_{s},F_{o},R,\gamma \right)$.

1. N is the number of UAVs. γ∈(0,1] is the reward discount factor.
2. $O=\{O_{1},\ldots ,O_{N}\}$ is the joint observation space, which consists of the observation state space $O_{i}$ of all UAVs.

Based on the UAV modeling in Section 2, it was assumed that each UAV had a Local State Field of View (LSFV) that corresponded to the obstacle detection range, i.e., $n_{o}$ cells. By extracting the target probability information of $n_{o}$ cells near the UAV’s location on the TEPM, the local state value of the LSFV was calculated at each time step *t*. The extracted cell uncertainty $u_{x,y}(t)$ was calculated as the Shannon entropy, serving as the local state value of the LSFV, as defined in Equation (8). Additionally, the local state value of the cell where an obstacle was detected was set to −1.

(8) $$u_{x,y}(t)=H[b_{x,y}(t)]=-b_{x,y}(t)\log_{2}b_{x,y}(t)-(1-b_{x,y}(t))\log_{2}(1-b_{x,y}(t))$$

In the METS mission, it was considered that the communication between individual UAVs would be unrestricted. Each UAV could obtain the current position information of other UAVs and the TEPM, aiding decision-making. Therefore, the observation state space $o_{i,t}$ of UAV i consisted of four parts, as defined in Equation (9):

(9) $$o_{i,t}=\{P_{t},D_{t},loc_{t,u},loc_{t,\bar{u}}\}$$

where $P_{t}$ is the TEPM at time t, $D_{t}$ is the LSFV of the UAV, $loc_{t,u}$ is the position of the UAV, and $loc_{t,\bar{u}}$ is the position of the other UAVs.

3. $A=\{A_{1},\ldots ,A_{N}\}$ is the joint action space, which consists of each UAV i choosing action $a_{i,t}\in A_{i}$ based on its own observed state space $o_{i,t}$.
4. S is the state space of the environment, $s_{t}\in S$ is the state of time slot t.
5. $F_{o}(s_{t},a_{t-1})=P(o_{t}=o|s_{t},a_{t-1})$ is the observation probability function.
6. $F_{s}(s_{t},a_{t})=P(s_{t+1}=s|s_{t},a_{t})$ is the state transition probability function.
7. R is the reward function, consisting of three components: an escape target search reward $J_{T}(t)$, an environment search reward $J_{E}(t)$, and a collision prevention reward $J_{C}(t)$. Weighting coefficients $\omega_{1}$, $\omega_{2}$, and $\omega_{3}$ were applied to balance these three components, as defined in Equation (10):
  (10) $$R(t)=\omega_{1}J_{T}(t)+\omega_{2}J_{E}(t)+\omega_{3}J_{C}(t)$$

The escape target search reward $J_{T}(t)$ was rewarded based on both the initial discovery of targets and the subsequent rediscovery of these targets. The search for an escape target in cell $C_{x,y}$ at time step t was evaluated as described in Equation (11). If the probability of a target’s existence in cell $C_{x,y}$ exceeded a threshold ε, the target was considered to be present in that cell.

(11) $$\beta_{x,y}(t)=\{\begin{array}{l} 1\;,b_{x,y}(t)>\varepsilon \\ 0\;,\;else \end{array}$$

The evaluation process for determining the existence of an escape target in a cell, initially and in subsequent evaluations, is represented by $\beta_{x,y}^{1}(t)$ and $\beta_{x,y}^{2}(t)$. Therefore, the escape target search reward was calculated as shown in Equation (12):

(12) $$J_{T}(t)=\sum_{x,y\in T_{1}}\beta_{x,y}^{1}(t)*r_{t1}+\sum_{x,y\in T_{2}}\beta_{x,y}^{2}(t)*r_{t2}$$

where $T_{1}$ and $T_{2}$ are the sets of locations where the escape target was initially discovered and subsequently rediscovered, respectively, and $r_{t1}$ and $r_{t2}$ are the reward factors for the initial discovery and subsequent rediscovery of the escape target, respectively.

The environment search reward $J_{E}(t)$ quantifies the extent of environmental exploration and is determined by the change in cell uncertainty $u_{x,y}(t)$ over time, as represented in Equation (13):

(13) $$J_{E}(t)=\sum_{x=1}^{N_{x}}\sum_{y=1}^{N_{y}}u_{x,y}(t)-u_{x,y}(t+1)$$

The collision prevention reward $J_{C}(t)$ was defined as the condition in which the distance between the UAV and an obstacle falls below a safe threshold, as represented in Equation (14):

(14) $$J_{C}(t)=r_{c}*\sum_{i=1}^{N_{u}}\sum_{k=1}^{N_{B}}1,\left\|B_{k}-u_{i,t}(x,y)\right\|\leq d_{safe}$$

where $r_{c}$ is the collision penalty factor.

### 3.2. Multi-UAV Escape Target Search Algorithm Based on MAPPO

In a multi-agent environment, the MAPPO algorithm [17] demonstrates superior adaptability and stability in strategy optimization compared to other MARL algorithms. To address the METS problem, we propose the multi-UAV Escape Target Search algorithm based on MAPPO (ETS–MAPPO), which includes two key components: first, the Spatio-Temporal Efficient Exploration (STEE) network to enhance the MAPPO algorithm’s capability to process spatio-temporal sequence information and explore the environment. Second, the Global Convolution and Local Ascent (GCLA) mechanism to overcome challenges posed by the variability of the escape target and the weak directionality of state inputs.

#### 3.2.1. Spatio-Temporal Efficient Exploration Network

The MARL algorithm focuses on both data utilization and environmental exploration when solving search problems. Utilization refers to the agent selecting the action that maximizes the reward from previous actions, while exploration involves the agent choosing a new action in anticipation of a higher reward. Balancing utilization and exploration poses a significant challenge in reinforcement learning [25], particularly in complex environments with multiple state inputs and sparse rewards.

In the MARL, empirical data contain both temporal and spatial information. Temporal information includes state dynamics, action continuity, and reward latency. Spatial information encompasses environmental complexity, relative UAV positions, and target distribution. Current approaches for data utilization commonly employ recurrent neural networks, such as LSTM [26] and GRU [27], for temporal data processing. Spatial data are primarily processed using convolutional neural networks, such as GCN [28] and HGCN [29]. These networks face challenges in relation to the efficient processing of both temporal and spatial information. As a solution, the Convolutional Long Short-Term Memory (ConvLSTM) [30] network, which combines convolutional operations with memory networks, has been introduced. The ConvLSTM network can capture both spatial and temporal features, thereby enhancing the performance of MARL algorithms. However, the ConvLSTM network lacks strong environmental exploration capabilities, necessitating improvements in this area. Currently, MARL algorithms employ epsilon-greedy and entropy regularization strategies for exploration, which introduce noise to the agents’ actions, but often result in low exploration efficiency. This paper proposes the use of the noise network, which adds parameterized noise to the neural network, thereby improving the exploration efficiency of MARL algorithms and preventing them from converging into local optima.

**Figure 4:**
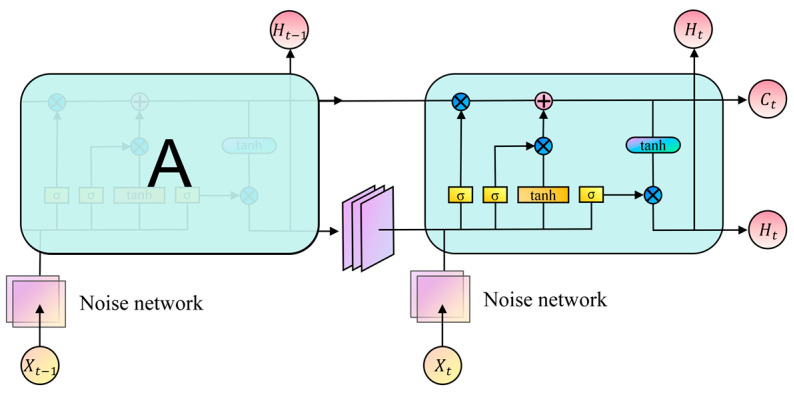
The STEE network architecture.

This paper proposes the STEE network architecture, which integrates the ConvLSTM network’s efficient processing of spatio-temporal sequential information with the noise network’s effective capability to conduct environmental exploration. The STEE network architecture is illustrated in Figure 4. Data were first normalized with features and then fed into the noise network to process state data features. The output from the noise network was then used as the input for the ConvLSTM network unit, and the final output of the STEE network was obtained through a multi-layer ConvLSTM network unit.

**Figure 5:**
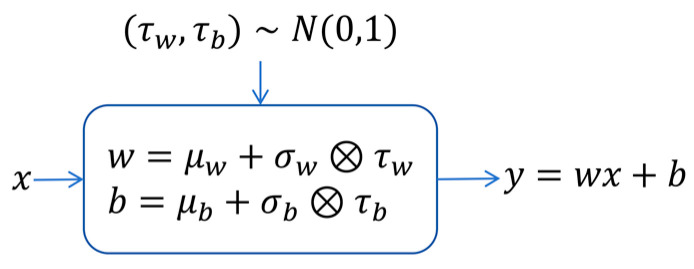
Network parameterized noise.

The ConvLSTM network unit was used to capture spatio-temporal data, enabling the modeling of dynamic environmental changes and long-term dependencies on historical information. The ConvLSTM network unit structure primarily comprises a forget gate, an input gate, a memory cell, and an output gate. The noise network is a fully connected neural network that incorporates parameterized noise, as depicted in Figure 5. It allows the STEE network to continuously learn the mean μ and variance σ of w,b during the training process.

#### 3.2.2. Global Convolution Local Ascent Mechanism

In the METS problem, the escape target initially remained stationary and only entered the escaping state after detecting the UAV. During the escaping state, the target took random actions, leading to positional changes and increased environmental instability. Under the existing mechanism, each UAV observes a larger number of state space parameters than the TEPM’s $N_{x}\times N_{y}$. However, these state parameters are sparse and lack clear directionality, offering no advantage when searching for the escape target. The UAV’s limited ability to capture changes in the escape target’s location hinders its ability to relocate the target, resulting in poor network convergence.

Currently, there is insufficient research on the poor convergence of networks due to target escape. This paper proposes the GCLA mechanism, which employs a global TEPM convolution operation and a target local area uncertain probability ascent operation. This mechanism enhances both the capability to capture escaping targets and the convergence of the algorithmic network.

**Figure 6:**
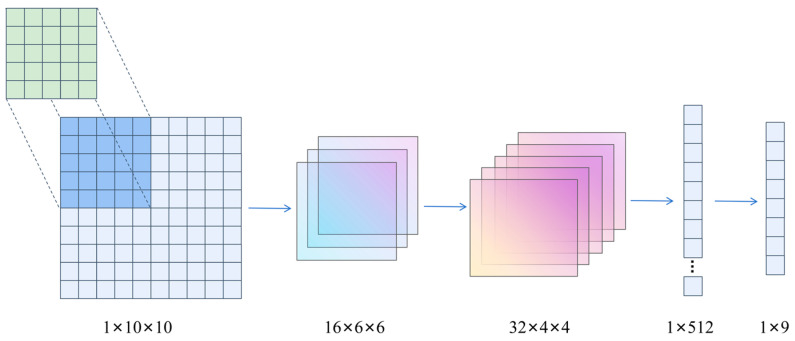
Processing of TEPM convolutional operations for 10×10 state parameters.

The global TEPM convolution operation optimizes the state parameters of the TEPM’s $N_{x}\times N_{y}$ into a set of values 1×9 through convolution. This operation aims to divide the entire task area into nine orientation zones to capture global direction guidance information. The TEPM convolution operation for the state parameters 10×10 is illustrated in Figure 6.

The target local area uncertain probability ascent operation involves increasing the uncertain probability of the target local area, after the initial search of the escape target. To enhance the UAV’s ability to re-search the area from which the target may have escaped, the operation increases the uncertain probability p(t) at a specific rate within the escaped area. This area is defined as the area spanning $n_{c}$ cells around cell $C_{x,y}$, as calculated in Equation (15). Subsequently, the TEPM is updated based on this revised uncertain probability within the escaped area.

(15) $$\left\{ \begin{array}{l} C(x^{\prime },y^{\prime })=C(x+i,y+i),i=[-n_{c},n_{c}]\\ p_{x^{\prime },y^{\prime }}(t)=p_{\max}+p_{x^{\prime },y^{\prime }}(t-1)e^{-\tau t} \end{array}\right.$$

where $p_{x^{\prime },y^{\prime }}(t)$ is the uncertain probability of target existence at $C_{x^{\prime },y^{\prime }}$ at time t and τ is the ascent rate.

After processing with the GCLA mechanism, the UAV’s observation state space $o_{i,t}$ is reformulated as shown in Equation (16):

(16) $$o_{i,t}=\{{P^{\prime }}_{t},D_{t},C_{t},loc_{t,u},loc_{t,\bar{u}}\}$$

where $C_{t}$ is the result following the convolution operation on the TEPM and ${P^{\prime }}_{t}$ is the updated TEPM.

### 3.3. ETS–MAPPO Algorithmic Framework

**Figure 7:**
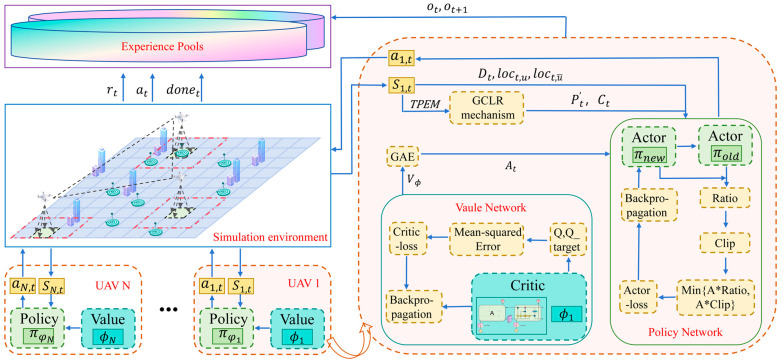
The ETS–MAPPO algorithmic framework.

The ETS–MAPPO algorithm builds upon the foundational architecture of MAPPO, incorporating the STEE network and the GCLA mechanism. As shown in Figure 7, the algorithm adopts the actor–critic network architecture and applies the centralized training and decentralized execution framework to solve the DEC-POMDP. Through interaction between the UAV and the simulation environment, empirical data are generated and stored in experience pools, from which the UAV extracts data to calculate the actor and critic network losses. The backpropagation algorithm is then employed to update these networks, enabling centralized training. Each UAV obtains observation state data from the environment and processes them through the GCLA mechanism. Based on the current actor network, these UAVs then select actions to interact with the simulation environment, thereby enabling decentralized execution.

The application paradigm of the ETS–MAPPO algorithm involves offline training and online execution. The pseudocode for the ETS–MAPPO algorithm is provided in Algorithm 1, below. The training phase focused on the optimization of both the actor and the critic networks. The actor network is designed to learn the mapping function $\pi_{\varphi }$ from the observed state space $O_{t}$ to the action $a_{t}$, and is constructed using a fully connected neural network. To avoid overmodification of the objective values, the actor network is implemented by optimizing a cropped alternative objective function $L_{t}(\theta )$, as shown in Equation (17):

(17) $$L_{t}(\theta )=E\{\min[r_{t}(\theta )A_{t},clip(r_{t}(\theta ),1-\varepsilon ,1+\varepsilon )A_{t}]+\sigma S[\pi_{\theta }(O_{t})]\}$$

where θ is the parameter of the actor network, $r_{t}(\theta )$ is the ratio of the new strategy to the old one, $A_{t}$ is the General Advantage Estimation (GAE), clip(⋅) is the cropping function, ε∈[0,1] is the truncation factor, $S[\pi_{\theta }(O_{t})]$ is the entropy of the strategy $\pi_{\theta }(O_{t})$, and σ is the hyper-parameter controlling the entropy coefficient.

The calculation of GAE is based on the Temporal Difference (TD) error $\delta_{t}$, as defined in Equation (18):

(18) $$\delta_{t}=r_{t}+\gamma V_{\phi }(s_{t+1})-V_{\phi }(s_{t})$$

where $r_{t}$ is the reward, γ is the discount factor, and $V_{\phi }(s_{t})$ is the value function of the critic network at time step t.

The GAE estimates the dominance function $A_{t}^{GAE(\lambda )}$ by considering the TD errors for k time steps forward from the current time step t and by performing a weighted summation of them. The specific weighted summation expression is provided in Equation (19):

(19) $$A_{t}^{GAE(\lambda )}=\left(1-\lambda \right)\left(\delta_{t}+\lambda \delta_{t+1}+\cdots +\lambda^{k-1}\delta_{t+k-1}\right)$$

where λ is the hyperparameter of TD error weights.

The purpose of the critic network, which is constructed using the STEE network, is to learn the mapping function $V_{\phi }$ from the state space $S_{t}$ to the real values. The critic network optimizes the network parameters using the mean square error loss function $L_{t}(\phi )$, with its expression provided in Equation (20):

(20) $$L_{t}(\phi )=E\{\max[{\left(V_{\phi }(s_{t})-{\hat{R}}_{t}\right)}^{2},{\left(clip(V_{\phi }(s_{t}),V_{\phi_{old}}(s_{t})-\varepsilon ,V_{\phi_{old}}(s_{t})+\varepsilon ,)-{\hat{R}}_{t}\right)}^{2}]\}$$

where ϕ is the critic network parameters and ${\hat{R}}_{t}$ is the discount reward.
**Algorithm 1:** ETS–MAPPO**Initialization:** The actor network parameters and the critic network parameters for each UAV and other relevant parameters.1. For episode = 1,2, …, M do.2. Reset environment state: x ← env.reset()3.      For time step = 1,2, …, T do4.           For UAV i = 1,2, …, N do5.                  Receive $P_{t},D_{t},loc_{t,u},loc_{t,\bar{u}}$
6.                  Obtain $o_{i,t}=\{{P^{\prime }}_{t},D_{t},C_{t},loc_{t,u},loc_{t,\bar{u}}\}$ by the GCLA mechanism7.                  Obtain the UAV action $a_{i,t}$ through $\pi_{i,\theta }(a_{i,t}|o_{i,t})$
8.           End9.           Execute actions $a_{t}$ and update the environment10.         Receive the environment reward $r_{t}$ and next state $o_{t+1}$
11.         Store trajectory $\tau +=[o_{t},r_{t},a_{t},o_{t+1}]$
12.      End13.      Compute advantage estimate $A_{t}$ via GAE on τ
14.      Compute the discount reward ${\hat{R}}_{t}$ and normalize15.      Split trajectory τ into chunks of length Y and store in experience pools16.      For mini-batch = 1,2,…,H do17.         *b* ← random sample mini batch from experience pools with all UAV data18.         Compute loss functions $L_{t}(\theta )$, $L_{t}(\phi )$ with data *b*
19.         Adam to update θ on $L_{t}(\theta )$
20.         Adam to update ϕ on $L_{t}(\phi )$
21.      End22. End

## 4. Experiments

To verify the effectiveness of the proposed ETS–MAPPO algorithm, it was compared against five classic MARL algorithms: MAPPO [17], MADDPG [31], MATD3 [32], QMIX [33], and IQL [34]. Ablation experiments were also conducted to demonstrate the contributions of the STEE network and the GCLA mechanism.

### 4.1. Simulation Environment and Parameter Settings

In the multi-UAV escape target search simulation scenario established, the environment was divided into two layers with a size of 2000 m × 2000 m. There were three UAVs, with a detection range for targets of 200 m and a detection range for stationary obstacles of 400 m. There were 10 escape targets, with a detection range for UAVs of 200 m, an escape range of 400 m, and a single escape attempt. The initial position of the target was taken to be randomly generated. The number of obstacles was 15. The simulation environment and network parameters are detailed in Table 1.

The simulation experiments were conducted using the following computer hardware and software: an Intel i5-12400F CPU manufactured by Intel Corporation and sourced from Hefei, China, 32 GB RAM, an NVIDIA GeForce RTX 4060Ti GPU manufactured by PC Partner and sourced from Dongguan, China, Python 3.11.5, and Pytorch 2.1.0.

### 4.2. Model Performance Analysis

The analysis of model performance began with the evaluation of the training results of each model, focusing on the convergence and performance metrics of six algorithm models: ETS–MAPPO, MAPPO, MADDPG, MATD3, QMIX, and IQL. Subsequently, the test results were analyzed to assess the generalization performance and real-time performance of the ETS–MAPPO algorithm model. Finally, the operational state of the ETS–MAPPO algorithm in the simulation environment at different time step was obtained. To ensure the reliability of the experiments, the number of network layers and neurons in each algorithm was kept consistent, and the algorithm parameters were appropriately tuned. All experiments were conducted three times under random seeds of 1, 2, and 3, and the average value of the three experiments was taken as the final experimental result.

#### 4.2.1. Analysis of Model Training Results

**Figure 8:**
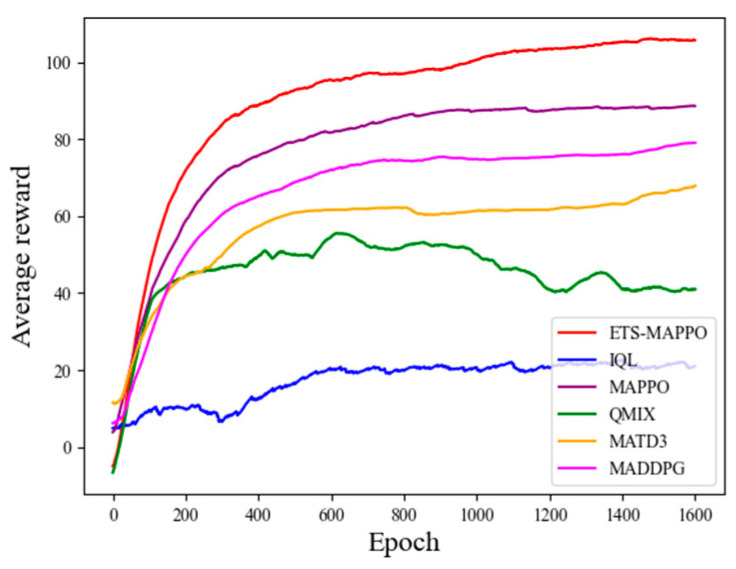
Comparison of the average reward values of the six algorithms.

Model training was conducted according to the experimental setup described above, with the convergence curves of the six algorithms—ETS–MAPPO, MAPPO, MAD-DPG, MATD3, QMIX, and IQL—presented in Figure 8. As the number of training rounds increased, the UAV average reward value gradually increased and converged. The average reward value curve of the ETS–MAPPO algorithm demonstrates a significant advantage over the other five classic algorithms.

**Figure 9:**
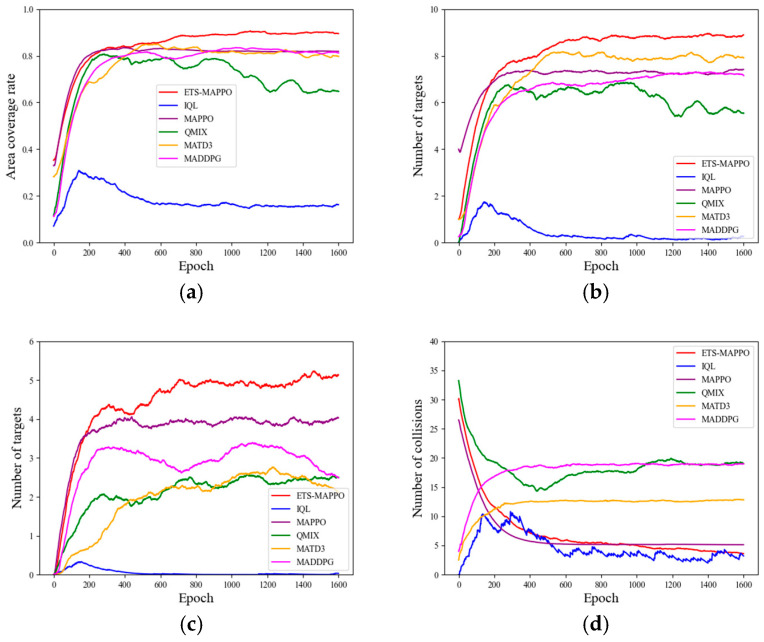
The specific parameter metrics train results. (**a**) Area coverage rate, (**b**) the number of initial target searches, (**c**) the number of target re-searches, (**d**) the number of collisions.

Besides the average reward results representing the combined effect of multiple metrics, the specific parameter metrics that measure the actual performance in the METS problem include the area coverage rate, the number of collisions, the number of initial target searches, and the number of target re-searches. As shown in Figure 9a–c, the ETS–MAPPO algorithm was compared with the other five algorithms in terms of area coverage, the number of initial target searches, and the number of target re-searches. Although the convergence curves are intertwined in the early stages, the results after convergence display clear advantages.

With regard to the indicator of the number of collisions shown in Figure 9d, the convergence trend of MADDPG and MATD3 differed from the downward convergence observed in the ETS–MAPPO, MAPPO, QMIX, and IQL algorithms by exhibiting upward convergence, leading to poorer results. Additionally, the results of the ETS–MAPPO, MAPPO, and IQL algorithms were similar.

The observed results can be attributed to the adoption of the STEE network and the GCLA mechanism within the ETS–MAPPO algorithm. These components enhance the processing of spatio-temporal sequence data and environmental exploration, strengthen the network state parameters, improve the algorithm’s learning efficiency, accelerate network convergence, and optimize the search for the escape target, ultimately yielding higher reward values, as reflected in the specific parameter metrics.

#### 4.2.2. Analysis of Model Testing Results

In order to further validate the experimental results, six algorithms, ETS–MAPPO, MAPPO, MADDPG, MATD3, QMIX and IQL, were tested. The model with the random seed of 1 was chosen for model testing, and each algorithm was tested over 20 rounds so as to take the average value as the test result; other environmental parameters were consistent with the model training above. Then, the number of targets and time steps were changed to verify the generalization performance of the ETS–MAPPO algorithm.

The model test results are shown in Table 2, which compares the performance of the six algorithms across five metrics. The ETS–MAPPO algorithm outperformed the others on four of these metrics: the average reward, the area coverage rate, the number of initial target searches, and the number of target re-searches. In terms of obstacle collisions, comparison of the training curves and other indicators revealed that the IQL algorithm experienced fewer obstacle collisions, likely due to its limited UAV exploration of the environment, rendering it less comparable. Therefore, excluding the IQL algorithm, the ETS–MAPPO algorithm surpassed the remaining four algorithms in this metric. To summarize, the ETS–MAPPO algorithm outperformed the other five classic algorithms across all five performance metrics and demonstrated superior performance overall.

**Figure 10:**
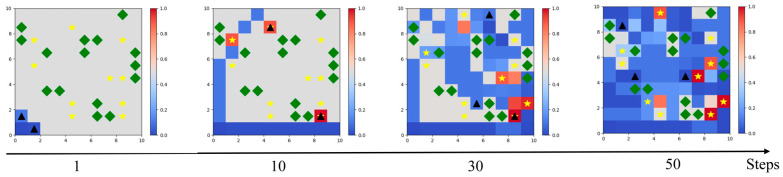
Results of UAV operation under 50 consecutive time steps in the simulation testing phase, where yellow pentacles represent targets, green diamonds represent obstacles, and black triangles represent UAVs.

Figure 10 gives the results of the environmental operation under 50 consecutive time steps in the test phase, at time step 1, time step 10, time step 30, and time step 50. At the beginning of the simulation, the UAV departed from the base and followed two paths for searching. From time step 1 to time step 10, it can be found that the UAV was trying to avoid collision with obstacles. From time step 10 to time step 30, it can be observed that the UAVs undertook a decentralized search and started searching for targets in full range, with many targets successfully searched for the first time. It can also be observed that some of the escaped targets had already escaped and there was a change in their position. From the 30th to the 50th time step, some targets were successfully searched for again. It can be observed that the UAV covered and searched most of the mission area. The above test results show that the ETS–MAPPO algorithm can reasonably and efficiently complete the area reconnaissance and escape target search.

**Figure 11:**
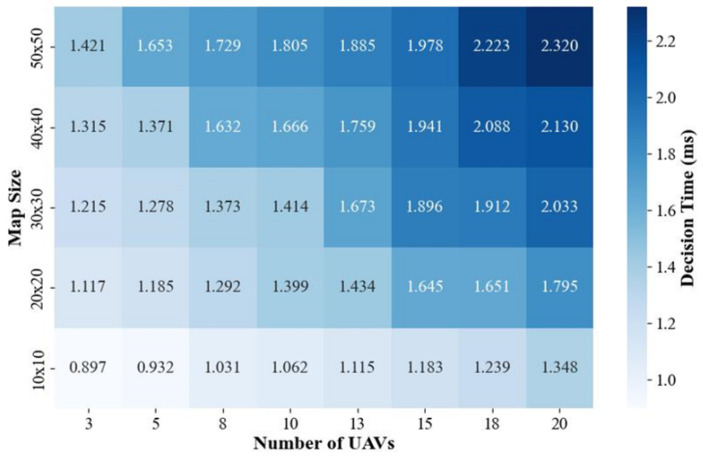
Results of the algorithm’s real-time performance test.

Figure 11 presents the results of the algorithm’s real-time performance test. From the results it can be seen that, as the number of UAVs increased and the range of the explored area expanded, the average decision time per UAV also increased. The variation in average decision time per UAV remained within the millisecond range (0.8–2.4 ms). In practice, this variation did not noticeably affect UAV operation.

To verify the generalization performance of the ETS–MAPPO algorithm, we varied the number of time steps and targets during testing. From Table 3 and Table 4, it can be seen that the average reward, the number of initial target searches, and the number of target re-searches increased with the increase in time steps and target count. The area coverage ratio remained near 0.9, and the percentage of initial repeat target searches ratio stayed around 0.5. This demonstrates that the ETS–MAPPO algorithm remained effective across different number of time steps and targets.

### 4.3. Ablation Experiment

To investigate the impact of the STEE network and the GCLA mechanism on the ETS–MAPPO algorithm’s performance, two variants were constructed: the GCLA–MAPPO, by removing the STEE network, and the STEE–MAPPO, by removing the GCLA mechanism. The test results for these algorithms across five performance metrics were analyzed. The specific configurations of the four algorithms in the ablation experiments are presented in Table 5, where “√” indicates inclusion and “×” indicates exclusion.

**Figure 12:**
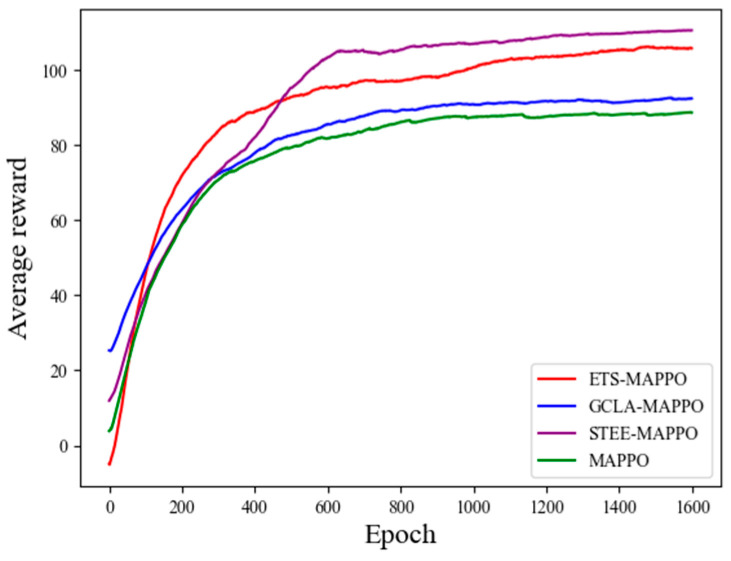
Comparison of average reward results from ablation experiment.

Figure 12 presents the training results of each ablation experiment algorithm in terms of average reward. From these results, it can be seen that the GCLA–MAPPO and MAPPO algorithms had similar reward values, while the ETS–MAPPO and STEE–MAPPO algorithms demonstrated higher reward values, with the ETS–MAPPO algorithm being slightly lower than the STEE–MAPPO algorithm.

Table 6 shows the test results of the four ablation experimental algorithms across five performance metrics. The results demonstrate that the ETS–MAPPO, STEE–MAPPO, and GCLA–MAPPO algorithms showed improvements over the MAPPO algorithm in four metrics: the average reward, the number of collisions, the number of initial target searches, and the number of target re-searches. However, on the area coverage rate metric, the GCLA–MAPPO algorithm was associated with a slightly lower value than that associated with the MAPPO algorithm.

These results can be attributed to the STEE network’s ability to efficiently process spatio-temporal sequence data and enhance environmental exploration, thereby improving the overall performance of the algorithms. Consequently, algorithms utilizing the STEE network achieved higher reward values. The GCLA mechanism caused the UAV to focus its search near the initial target discovery area, which often includes regions that have already been explored. Given the limited number of time steps, the UAV lacked sufficient time to search unexplored areas after repeatedly scanning near the initial target area. This led to a decrease in the area coverage rate and an increase in the number of target re-searches. These findings suggest that the STEE network and the GCLA mechanism effectively enhance the performance of the ETS–MAPPO algorithm in the METS problem.

## 5. Conclusions

With regard to the multi-UAV escape target search task, this paper addresses the challenges associated with enabling escape target search and efficiently utilizing the sample data from the MARL algorithm, particularly in relation to maintaining a balance between utilization and exploration. This paper proposes a multi-UAV escape target search algorithm that combines the STEE network with the GCLA mechanism, built upon the MAPPO algorithm and applied to the multi-UAV escape target search task. Experimental results demonstrate that the ETS–MAPPO algorithm excels in addressing the METS problem, outperforming the other five MARL algorithms across all metrics. Ablation experiments confirm that the STEE network enhances the utilization of spatio-temporal sequence data while effectively balancing environmental exploration, thereby improving the algorithm’s overall performance. Additionally, the GCLA mechanism significantly improves performance in the escape target search problem.

Future work will investigate the performance of our proposed ETS–MAPPO algorithm in larger-scale scenarios involving more UAVs, while continually optimizing the training efficiency of the network model.

## Figures and Tables

**Table 1:** The simulation environment and network parameter settings.

Parameter	Value
Total running steps	2,000,000
Time step per round	50
Actor network learning rate	5 × 10^−4^
Critic network learning rate	5 × 10^−4^
Hidden layer size	64
Discount factor	0.99
Detection payload correct rate	0.90

**Table 2:** Six algorithms test results.

Algorithms	Average Reward	Area Coverage Rate	Number of Collisions	Number of Target Initial Searches	Number of Target Re-Searches
IQL	24.85	0.15	2.68 *	0.55	0.09
QMIX	41.95	0.63	18.11	5.25	2.43
MADDPG	79.73	0.80	18.95	6.93	2.18
MATD3	71.11	0.78	12.67	7.87	1.80
MAPPO	88.86	0.82	5.12	7.45	2.75
ETS–MAPPO	106.21	0.89	3.18	9.09	5.11

* indicates that the data is not compared with other data.

**Table 3:** Experimental test results for changing the number of time steps.

Time Steps	Average Reward	Area Coverage Rete	Number of Collisions	Number of Target Initial Searches	Number of Target Re-Searches
50	106.21	0.89	3.18	9.09	5.11
60	111.65	0.96	9.70	9.55	5.65
80	124.86	0.99	16.20	9.73	7.05
100	129.76	1.00	18.4	9.82	7.10

**Table 4:** Experimental test results of changing the number of targets.

Target Numbers	Average Reward	Area Coverage Rate	Percentage of Initial Repeat Target Searches	Number of Target Initial Searches	Number of Target Re-Searches
5	102.26	0.94	0.51	4.55	2.33
10	106.21	0.89	0.57	9.09	5.11
15	107.51	0.92	0.48	13.05	6.35

**Table 5:** Ablation experimental setup.

Algorithms	STEE Network	GCLA Mechanism
ETS–MAPPO	√	√
STEE–MAPPO	√	×
GCLA–MAPPO	×	√
MAPPO	×	×

√ indicates that the network or mechanism is used. × indicates that the network or mechanism is not used.

**Table 6:** Results of ablation experiment.

Algorithms	Average Reward	Area Coverage Rete	Number of Collisions	Number of Target Initial Searches	Number of Target Re-Searches
MAPPO	88.86	0.82	5.12	7.45	2.75
STEE–MAPPO	109.28	0.93	0.36	9.85	3.05
GCLA–MAPPO	89.98	0.77	0.84	8.89	4.23
ETS–MAPPO	106.21	0.89	3.18	9.09	5.11

## Data Availability

The data presented in this study are available upon request from the corresponding author.
